# Pollution of Beach Sands of the Ob River (Western Siberia) with Microplastics and Persistent Organic Pollutants

**DOI:** 10.3390/jox14030055

**Published:** 2024-07-25

**Authors:** Yulia A. Frank, Yulia S. Sotnikova, Vasiliy Yu. Tsygankov, Aleksey R. Rednikin, Maksim M. Donets, Elena V. Karpova, Maksim A. Belanov, Svetlana Rakhmatullina, Aleksandra D. Borovkova, Dmitriy N. Polovyanenko, Danil S. Vorobiev

**Affiliations:** 1 Biological Institute, Tomsk State University, 36 Lenin Ave., 634050 Tomsk, Russiadanilvorobiev@yandex.ru (D.S.V.); 2 N.N. Vorozhtsov Institute, Organic Chemistry, Siberian Branch, Russian Academy of Sciences (SB RAS), 9 Acad. Lavrentiev Ave., 630090 Novosibirsk, Russia; sotnikova@nioch.nsc.ru (Y.S.S.); karpovae@nioch.nsc.ru (E.V.K.); dpolo@nioch.nsc.ru (D.N.P.); 3 Pacific Geographical Institute, Far-Eastern Branch, Russian Academy of Sciences (FEB RAS), 7 Radio Street, 690041 Vladivostok, Russia; tsig_90@mail.ru (V.Y.T.);

**Keywords:** riverine microplastics, Siberian rivers, beach sands, polychlorinated biphenyls (PCBs), organochlorine pesticides (OCPs)

## Abstract

Microplastics (MPs) in aquatic environments can be associated with various substances, including persistent organic pollutants, which add to the problem of plastic ecotoxicity. The abundance of 1–5 mm microplastics and concentrations of particle-adsorbed organochlorine pesticides (OCPs) and polychlorinated biphenyls (PCBs) in sandy sediments from three beaches in recreational areas along the upper Ob River in Western Siberia were assessed. MP pollution levels in the Ob River beach sands ranged from 24 ± 20.7 to 104 ± 46.2 items m^−2^ or, in terms of mass concentration, from 0.26 ± 0.21 to 1.22 ± 0.39 mg m^−2^. The average abundance of MP particles reached 0.67 ± 0.58 items kg^−1^ or 8.22 ± 6.13 μg kg^−1^ in the studied sediments. MP concentrations were significantly higher in number (*p* < 0.05) and mass (*p* < 0.01) at the riverbank site downstream of the Novosibirsk wastewater treatment plant (WWTP) outfall compared to these at the upstream and more distant beaches. Most MPs (70–100%) were represented by irregularly shaped fragments. The polymer composition of MPs varied between sites, with a general predominance of polyethylene (PE). The study revealed associations of MPs with PCBs and OCPs not previously detected in the riverbed and beach sediments, suggesting that these substances are circulating in the Ob River basin. Although MP concentrations were higher downstream of the WWTP, the maximum levels of particle-associated OCPs were observed in the beach sands of the site farthest from the urban agglomeration. The pesticides γ-HCH, 4,4-DDT, and 4,4-DDE were detected on MPs at relatively low concentrations. PCBs were more abundant in the studied samples, including 118 dioxin-like congener. The results obtained indicate that the Ob River is susceptible to plastic and persistent organic pollutant (POP) contamination and serve as a starting point for further studies and practical solutions to the problem.

## 1. Introduction

Large rivers play an important role in the global cycle of microplastics <5 mm (MPs), as they are closely linked to the terrestrial environment and provide important pathways for the transport of pollutants, including the smallest plastic particles [1,2,3,4]. The abundance of MPs in river waters worldwide varies from several items in 1000 m^−3^ to over 1 million items m^−3^ [5,6,7,8]. Due to their ubiquity, longevity, and abundance, plastic debris, in particular MPs, have been described as a new type of sediment particle and therefore a new type of river sediment load [9,10,11]. Although plastic debris enter freshwater ecosystems through a variety of ways, such as sewage discharge, storm and flood runoff, and mismanaged plastic waste [12,13,14], industrial and municipal wastewater discharge is one of the major sources of MPs in rivers [15]. Wastewater treatment plants (WWTPs) are currently not designed to remove MPs completely, leading to environmental pollution as millions of particles are released into receiving waters every day [16,17,18,19]. In regions where WWTPs have a high efficiency of MP removal (up to 97–99%), waste water still significantly contributes to pollution because of the large volumes treated [12,20].

The specific properties of MPs allow them to persist in the aquatic environment, to be transported over long distances, to be redistributed between river ecosystem compartments, and to bioaccumulate in food chains up to humans [21,22,23]. One of the issues of serious concern in the ecotoxicology of aquatic MPs is their interaction with various toxic compounds in natural ecosystems. Metals and persistent organic pollutants (POPs), such as polycyclic aromatic hydrocarbons (PAHs), polychlorinated biphenyls (PCBs), dioxins, and other organochlorine pesticides (OCPs), are adsorbed and concentrated on the surface of MP particles from the surrounding water due to hydrophobic partitioning [24,25]. Of all the substances associated with MPs in the aquatic environment, POPs are of particular concern because of their long-term persistence and harmful properties to most living organisms [26]. POPs are recognised to have toxic properties, resist degradation, and are transported through water and transferred in living systems [27,28].

A number of studies suggest that tiny plastic particles can act as sinks and transporters of POPs in natural waters and food webs [5,22,29,30,31,32]. This may be particularly important in continental freshwaters, such as rivers, lakes, and reservoirs, where concentrations of both MPs and bioaccumulative toxic substances are expected to be higher than these in marine systems [22,33,34]. However, studies aimed at screening river contamination with MPs and associated organic pollutants are still rare [8]. The study of riverbank sediment contamination is important in this regard, as beaches are an interface where humans can come into direct contact with MPs and adsorbed toxicants [35].

The microplastic contamination of riverbanks and floodplains can originate from terrestrial sources, such as local litter fragmentation, or from river transport [14,36,37,38]. MPs entering river systems not only float in the water but are redistributed between bottom sediments, river banks, floodplains, estuaries, etc., as hydrological, meteorological and biogeochemical variables mobilise, transport and deposit plastics in different river compartments [13,39,40,41]. The retention mechanisms and residence times of MPs on river banks and floodplains remain largely unknown, but it is clear that sedimentation rates and river hydrological characteristics play an important role [13,42]. Flood events caused by extreme precipitation or snowmelt have a major impact on the transport and settlement of MPs in freshwater ecosystems [43]. Floods deliver high loads of MPs from terrestrial sources to the river, while simultaneously exporting remobilised MPs from the bed sediment downstream or overbank, where they are deposited on beach sediments and floodplains [44]. The redistribution of MPs and associated toxic chemicals between water, bottom sediments, and river banks may be very pronounced in the North Eurasian rivers due to their predominantly snow-fed nature and close relationship between solute and solid transport and water level fluctuations, as has been estimated for the Ob River [45,46].

The Great Siberian rivers are under-studied for plastic and POP pollution, despite their length, importance to local populations, and potential contribution to Arctic seas. The preliminary studies of MP loads in the Ob, Yenisei, and their tributaries have been published, focusing on surface water, bottom sediments, and fish [47,48,49]. In this study, we investigated the beach sediments of the Ob as part of the river ecosystem and with which local people are in direct contact. To our knowledge, there are no published data on the abundance of MPs in the beach sediments of the Siberian rivers, including the Ob, together with information on adsorbed POPs. The aim of the current study was to access the MP pollution and evaluate the associated POPs, namely OCPs and PCBs, in the beach sands of the Ob River in the recreational areas around the city of Novosibirsk, Western Siberia.

## 2. Materials and Methods

### 2.1. Sampling Sites

**Figure 1:**
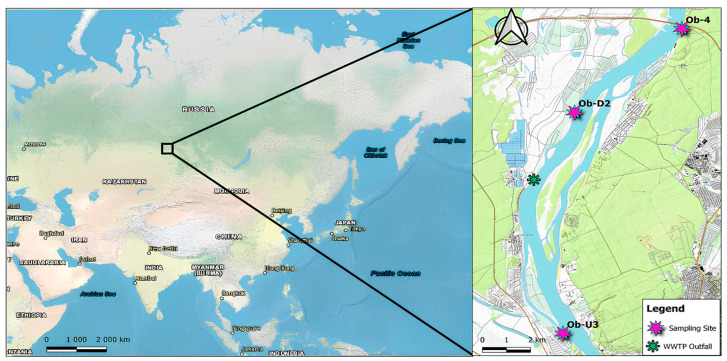
Schematic map of Asia and location of the sampling sites (created using QGIS 3.18).

Three sites along the upper Ob River in Western Siberia (Russia) were sampled in late summer 2022 (August 24) to investigate MP pollution and possible POP associations. All three recreational beach sites were located on the Ob banks with a total transect length of ~16.5 km below the city of Novosibirsk with a population of 1635 thousand (Figure 1). The sampling sites, arranged from upstream to downstream of the river, were represented by recreational areas and included the following: (1) Ob-U3, beach of the cottage village within the city of Novosibirsk, 7 km upstream of the outfall of the Novosibirsk WWTP, left bank, 55°03′29.6″ N, 82°48′13.4″ E; (2) Ob-D2, beach of the cottage village near Kudryashovsky settlement, Novosibirsk region, 4 km downstream of the WWTP outfall, left bank, 55°09′19.1″ N 82°49′00.2″ E; (3) Ob-4, beach of the recreation centre near the Mochishche settlement, Novosibirsk region, 9.5 km downstream of the WWTP outfall, right bank of the Ob, 55°11′08″ N 82°53′00″ E.

### 2.2. Sediment Sampling and Processing

Sand samples were collected 5 m from the water’s edge using an adapted sampling frame method [50]. A stainless steel frame with an area of 625 cm^2^ (25 × 25 cm) and a clean stainless steel scoop were used for sampling. The depth of the frame was 5 cm, as it is known that more than half of the MPs are typically found in the upper 5 cm of beach sediments [35,51]. For each site, sand from 4 randomly spaced 625 cm^2^ sample areas (0.25 m^2^) was successively sieved through standardised metal sieves with pore diameters of 5 mm and 1 mm [52]. Between 4919 and 5006 g of sand (~20 kg per site) were collected and sieved from each of the 625 cm^2^ areas within the sampling sites. MP-like particles were removed from the sieves with tweezers and placed in glass Petri dishes for further laboratory analysis. In this way, 12 sets of particles were obtained from three beaches: Ob-U3, Ob-D2, and Ob-4. To control for the possible field contamination of the samples, Petri dishes with wet clean filter paper were used, which were opened during sampling, as described in [53]. A total of 12 blanks (*n* = 4 at each sampling site) were collected and analysed in the laboratory together with the samples.

### 2.3. Microplastic Quantification and Characterisation

Particles collected from the beach sediments of the Ob River were examined by light microscopy. A Micromed MS-5-ZOOM trinocular light stereomicroscope (Shenzen, China) equipped with a ToupCam UA1000CA 10.0 MP video eyepiece (Zhejiang, China) was used for visual analysis and MP counting. For each particle, the size along the largest axis was determined to confirm that it belonged to MPs smaller than 5 mm. The minimum MP size in our study was 1 mm due to sampling limitations. The mass of plastic particles in a sample was determined using an OHAUS Explorer microbalance (Parsippany, NJ, USA) with an accuracy of ±0.001 mg. The direct microscopy of the blanks (*n* = 12) using a Micromed MS-5-ZOOM trinocular light stereomicroscope revealed no particles in the analysed size range of 1–5 mm.

Varian 640-IR FT-IR spectrometer (Palo Alto, CA, USA) equipped with the PIKE MIRacle™ ATR accessory with single reflection diamond crystal was used to obtain the spectra of the collected MPs and identify the polymer composition. Spectra were collected at a resolution of 4 cm^−1^ with 64 scans in the 4000–400 cm^−1^ range. Identification of the polymers was carried out using IR spectra databases accumulated at the N.N. Vorozhtsov Institute of Organic Chemistry (Novosibirsk, Russia). A total of 67 particles were analysed by FT-IR, of which 61 were identified as plastics and 40 as MPs ≤ 5 mm after precise measurement.

MP concentrations in sand were expressed in various units (items/mg m^−2^, items/mg m^−3^) and calculated as items/mg kg^−1^ using dry sand sample densities of 1.574–1.602 g cm^−3^ to allow comparisons with other published results. The dataset from each of the three sampled sites included MP abundances obtained for four subsamples from 0.25 m^2^ sample areas, 12 sets in total. Datasets in all units (items per square, volume, and dry weight) from different sites were compared using non-parametric tests due to the small sample sizes (*n* = 4). Data analysis was performed with R statistical software (v4.0.5) [54]. The Mann–Whitney U-test was used to access the differences in MP number and mass concentrations between sampling sites, with the results considered statistically significant at *p* ≤ 0.05.

### 2.4. Assessment of Persistent Organic Pollutant Concentrations

Pooled MPs from each sampling site were treated with n-hexane, followed by cleaning with concentrated sulphuric acid for the extraction of OCPs, as described in [55]. Concentrations of OCPs (isomers of HCH (α-, β-, γ-HCH), DDT, and its metabolites (DDD, DDE), and polychlorinated biphenyls (PCBs), including 28, 52, 155, 101, 118, 143, 153, 138, 180, 207 PCBs) in the samples were measured by gas chromatography–mass spectrometry (GC-MS).

Analysis was performed on a Shimadzu GCMS-QP2010 Ultra gas chromatograph mass spectrometer (Kyoto, Japan) equipped with an AOC-5000 auto-injector and an SLB-5ms capillary column, using helium as the carrier gas at a constant flow rate of 1 mL min^−1^. The injector and detector temperatures were set to 250 °C and 150 °C, respectively. The oven temperature was programmed as follows: 100 °C for 4 min, 7 °C min^−1^ to 310 °C and 6 min hold at 310 °C. A 2 µL sample was injected in splitless mode with the split outlet opened after 1 min. The mass spectrometer was operated in electron ionisation (EI) mode. An SIM program was written for GC-MS acquisition and quantification. The three most abundant ions were monitored for each substance. The retention time, mass, and relative abundance of the confirmation ions to the target ion were used as identification criteria. A relative (percentage) uncertainty of less than ±20% of the theoretical value was considered acceptable. Peak areas were measured using the GCMS Postrun Analysis program available in the Shimadzu Workstation GCMS Solution software, Version 4.20 [56].

Laboratory blanks were extracted and analysed. The retention times for the standard samples were constant, and we therefore relied on component identification. Standard POP solutions by Dr. Ehrenstorfer (Teddington, Middlesex, UK) and AccuStandard (New Haven, CT, USA) in the concentration range of 0.1–1000 ng ml^−1^ were used to identify specific substances. The calibration lines showed excellent linearity over the studied concentration range. To assess the quality of the method, a recovery study was performed using the standard addition methods. Ten samples of plastic pellets were spiked with a mixture of OCP and PCB standards. The spiked samples were extracted and analysed as described above. The results showed that the mean recoveries ranged from 84.6 to 99.5%, and relative standard deviation ranged from 4.6 to 10.1%. This indicates that the analytical procedures described for the determination of POPs in this study were reliable, reproducible, and efficient. The limits of detection (LODs) were calculated as 3 × SD (standard deviation of 10 samples) of the PCB and OCP levels in the procedural blanks. For analytes not detected in the procedural blanks, the LOD was calculated as the amount of analyte per sample corresponding to the lowest calibration standard [56].

## 3. Results

### 3.1. Quantification and Characterisation of Microplastics in Ob River Beach Sediments

A total of 16 suspected MP particles were sampled at site Ob-U3, 44 particles were sampled at site Ob-D2, and seven particles were sampled at site Ob-4. Based on spectroscopic analysis and precise size determination, 50–86% of the particles were classified as MPs (<5 mm). In total, 8 plastic particles > 1 mm were identified and selected for further analysis in 4 samples from site Ob-U3, 26 particles were identified and selected for further analysis in samples from site Ob-D2, and 6 particles were identified and selected for further analysis in samples from site Ob-4 with total masses of 120, 304, and 65.2 µg, respectively (Table 1). The MP contamination levels in the Ob beach sands ranged from 24 ± 20.7 to 104 ± 46.2 items m^−2^ (mean 53.3 ± 46.6) or 480 ± 413 to 2080 ± 924 items m^−3^ (mean 1067 ± 929) by volume. Mass concentrations varied from 0.26 ± 0.21 mg m^−2^ and 5.22 ± 4.1 mg m^−3^ at site Ob-4 to 1.22 ± 0.39 mg m^−2^ and 24.3 ± 7.73 mg m^−3^ at the most contaminated site Ob-D2. The number of MP particles per 1 kg of dry sand from the Ob beaches with a density of 1.57–1.60 g cm^−3^ ranged from 0.3 ± 0.26 to 1.31 ± 0.58 items (3.26–15.2 µg) in the samples studied, with a mean of 0.67 ± 0.58 items kg^−1^ and 8.22 ± 6.13 µg kg^−1^, respectively (Table 1). MP concentrations were significantly higher in number (*p* < 0.05) and mass (*p* < 0.01) at the site Ob-D2 downstream of the Novosibirsk WWTP outfall than at upstream site Ob-U3 and more distant site Ob-4. No differences in the MP number or mass were observed between the Ob River beach sands at sites Ob-U3 and Ob-4.

**Figure 2:**
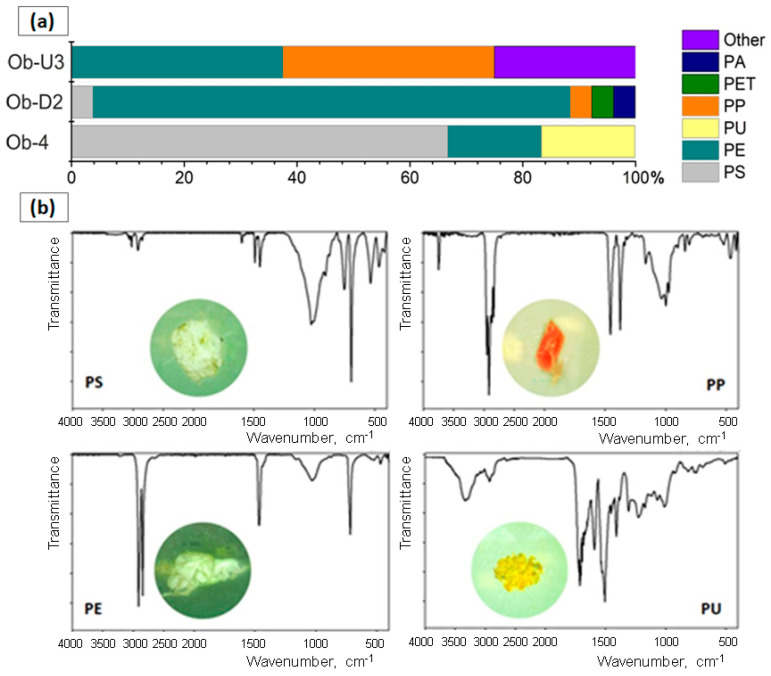
Polymer composition of MPs recovered from the Ob River beach sediments (**a**); FT-IR spectra of the selected particles corresponding to PS, PP, PE, and PU (**b**).

MPs found in the Ob beach sediments were represented by different polymers such as polyethylene (PE), polyamide (PA), polypropylene (PP), polystyrene (PS), polyethylene terephthalate (PET), and polyurethane (PU) (Figure 2). The percentage of different polymer types varied between the three sites. PE was found in all samples and accounted for up to 85% of the total MP amount at site Ob-U2 (Figure 2a). The remaining 15% of the particles from site Ob-U2 consisted of PS, PP, PET, and PA. Particles recovered from the site Ob-D3 were equally dominated by PE and PP (37.5% each), and contained other polymers represented by copolymers PE and PP, making up a total of 25%. PS was the most prevalent polymer among MPs found at site Ob-4 (67%), followed by PU and PE (Figure 2a). Figure 2b shows the FT-IR spectra of some collected MPs.

**Figure 3:**
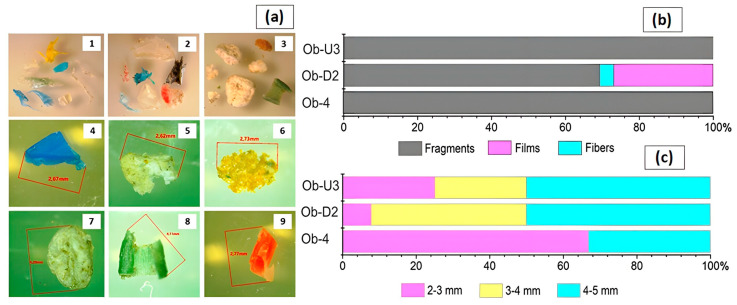
MPs from the Ob River beach sediments (**a**): irregularly shaped fragments, fibre, and films from Ob-D2 (1–2) and Ob-4 (3) samples; fragments from Ob-U3 (4, 9) and Ob-4 (5–8) samples; distribution of MPs recovered from the sediments by shape (**b**) and size (**c**).

Microplastics ranging from 2 to 5 mm in the largest size (from a minimum of 2.25 mm to a maximum of 4.97 mm) and of various shapes and colours were recovered from the beach sand of the Ob River (Figure 3). Half of all the beach sand MPs at sites Ob-U3 and Ob-D2 were represented by particles of 4–5 mm, while the majority of the particles at the more distant site Ob-4 (66.7%) belonged to 2–3 mm size class. The lowest abundance of 2–3 mm MPs was found at site Ob-D2, which accounted for 7.7% of the total amount of MPs. No particles between 1 and 2 mm identified as plastics were observed despite the use of a 1 mm mesh sieve for sampling. The particles of 1–2 mm (green fragment of 1.37 mm and white fibre of 1.7 mm) were recovered from the Ob beach sand at site Ob-3D; these were not identified as plastics by FT-IR analysis, but as silicate and cellulose, respectively. Most MPs were represented by irregularly shaped fragments, which accounted for 100% of all particles found at sites Ob-U3 and Ob-4, and 70% of MPs from the beach sediments at site Ob-D2. An elongated particle classified as ‘fibre’ and thin PE films were recovered from the beach sediments, only at site Ob-D2 downstream of the WWTP outfall (Figure 3b).

### 3.2. Persistent Organic Pollutants Associated with Microplastics in Ob River Beach Sediments

MPs collected at each of the three study sites were pooled for organic pollutant analysis. Different OCPs and PCBs were identified in all pooled MP samples, which varied from site to site (Table 2). HCH was only detected in sample Ob-D2, collected downstream of the WWTP, and was represented by the γ-isomer, whose concentration reached 25.6 ng g^−1^ or 0.04 ng per MP item. In the samples Ob-D2 and Ob-4, DDT and its metabolites were represented by the 4,4′-forms, DDT and DDE, respectively. The concentration of the former was 2.5 µg, and that of the latter was 3.44 µg per gram of MPs, making an average of 4.3 and 7.6 ng item^−1^, respectively (Table 2).

Polychlorinated biphenyls were represented in all three samples by two congeners, 118 and 180 PCBs, accounting for up to 1.27 and 3.48 in µg g^−1^ MPs from site Ob-4. The MP sample from this site also contained PCB 28 at a concentration of 739 ng g^−1^ or 1.6 ng item^−1^. PCB 153 was only detected in the sample Ob-D2 accounting for 652 ng g^−1^ with an average of 1.1 ng per MP item (Table 2).

## 4. Discussion

A few authors have focused on the MP pollution of river banks, but the abundance of MPs in freshwater bottom sediments is being actively studied around the world [2,57,58,59]. For rivers, it is quite difficult to clearly distinguish between beach and bottom sediment contamination due to the significant changes in water levels during the different phases of the hydrological regime. The abundance of MPs in the river sediments of 30.3 ± 15.9 items kg^−1^ was found to be one of the lowest in the world, but small fibres less than 1 mm are predominant in freshwater sediments [59]. At the same time, MP accumulation depends on many factors other than anthropogenic load, such as flow and sedimentation rates and the nature of bottom sediments. It is very important to take into account the range of detectable particle sizes when comparing quantitative data on MP distribution.

The average number of 0.15–5 mm MPs in the bottom sediments of the Siberian river Nizhnyaya Tunguska, the longest tributary of the Yenisei, reached 543 ± 94.1 particles kg^−1^ according to the preliminary assessment [48]. For the Ob River basin, only a preliminary quantitative assessment of MP abundance in surface water is available [47,60]; it shows the presence of variously shaped plastics of 0.15–5 mm, predominantly irregular fragments and fibres of 0.3–1 mm. Due to seasonal changes in water levels, the Ob beach sands sampled in the current study belong to river sediments, which allows comparisons with the published results on both beach and river bottom MPs. The number of “large” MPs of 1–5 mm per kg of Ob beach sand estimated in August 2021 varied from 0.3 to 1.31 items (mean 0.67 ± 0.58) or 3.26 to 15.2 (8.22 ± 6.13) μg in the sediments studied (Table 1). This is much lower than the values obtained for the bottom sediments of the Arno River in Italy (average 1760 items kg^−1^) [61], the Amazon River (up to 8178 items kg^−1^) [62], as well as the sandy beaches of the Mississippi reservoir in the USA, which ranged on average from 270 to 950 items kg^−1^ [63]. However, the authors used different methods to extract and identify MPs, and quantitative assessments included particles of different sizes, often < 1 mm and in some papers up to several microns, and in many cases, did not provide information on the mass of MPs in the samples. This makes comparisons difficult. For example, in assessing the MP content in the Solimões, Negro, and Amazon rivers, Gerolin et al. extracted and identified particles from 63 µm to 5 mm and reported their high absolute concentrations, but did not measure the mass [62]. In the study by Gao et al. [63], MPs were recovered from sand samples by wet density separation in a rich ZnCl_2_ solution, the minimum size was limited by a 45 µm mesh screen, and the number of MPs increased dramatically as the size decreased. The inconsistency of sampling and processing methods for river sediments in Asia, which complicates data analysis, has been highlighted previously [64]. When compared to similarly sized MPs in marine beach sands, the Ob River bank is less contaminated. The median contamination level of a sandy tide-less coast of the Curonian spit national park, the Baltic Sea, with 0.5–2 mm MPs, was 46 ± 22 items kg^−1^ [65]. The abundance of 0.5–5 mm MPs in the beach sand of the Can Gio coast, sampled near Ho Chi Minh City (Vietnam), reached a maximum of 4.49–6.58 items or 13.3–18.7 mg kg^−1^ [66]. Gallitelli et al. [67] sampled both sandy gravel sediments from both the bed and banks of the Mignon River in Italy following a linear transept. The mean concentration of micro- and mesoplastics in the bank sediments of the Mignon was 1.16 items kg^−1^, ranging from 0 to 2.45, of which 86.4% were MPs of 1–5 mm, comparable to our results. In general, the level of MPs in the Ob is comparatively low in relation to other river systems. This may be due to the fact that Western Siberia is a remote area along the 5410 km of the Ob and, with the exception of Novosibirsk (1635 thousand), Barnaul (635.5 thousand), Surgut (396 thousand), and a few other cities, it is sparsely populated, especially when compared to river systems in more urbanised regions of the world. The study shows that MP pollution is a global problem, relevant even in remote areas such as Western Siberia and, as previously shown, Eastern Siberia [48].

MP particles found in the Ob River beach sediments are predominantly represented by irregularly shaped fragments, indicating their secondary origin from larger plastic debris, but their sources can be variable. The abundance of MPs may be related to the WWTP contribution as one of the possible factors. In the beach sand of site Ob-D2, downstream of the Novosibirsk WWTP outfall, the number and mass of MPs were significantly higher compared to the upstream site Ob-U3 or more distant site Ob-4, *p* < 0.05 and *p* < 0.01, respectively. MPs found at site Ob-D2 were more diverse in terms of polymer composition and shape, including films and fibres, as well as irregularly shaped fragments of 2–5 mm. Although WWTPs are assumed to mainly discharge the finest MPs due to insufficient treatment, the relatively coarse particles of 1–5 mm can also make up a significant proportion of the effluent. For example, MPs of this size class accounted for up to 8.3% of total plastic particles in the effluent of the Cádiz sewage treatment plants in Spain [68] and 39.4–40.5% of 220,000–1.5 million particles per day in Adana, Turkey [69]. Earlier maritime ports and bad beach management but not WWTPs were recognised as sources of coastal MP pollution along the coast of Asturias, southwest Bay of Biscay, Spain [70]. However, it may be a significant factor in regions with a higher fluvial contribution to coastal pollution, as recently recognised [66,71]. It is known that WWTPs around the world treat domestic sewage and urban storm water, but do not completely remove MPs and discharge them in concentrated form into watercourses at specific points [17,18,19]. The Novosibirsk WWTP was built in the 1980s, and after recent modernisation, its capacity is about 600,000 m^3^ day^−1^ [72], posing a concern in terms of the MP pollution of the Ob River. Sewage treatment plant effluents are a major contributor to MP in rivers and coastal waters in China, South Korea, Southwest Asia, and the rest of Asia [64,73,74,75]. However, their absence in some cases leads to even greater pollution, as has been shown for several Asian watercourses [64,76].

Potential sources of OCPs associated with MPs in the Ob beach sand are of interest. The composition of the organic component of the bottom sediments of the Ob River in its middle course was studied in [77]. An abundance of PAHs was observed in river sediments downstream of major tributaries such as the Tom and Irtysh rivers, suggesting their origin from industrial and oil-producing regions in the Ob Basin. Industrial sources were identified as the most important source of organic pollutants in the bottom sediments of the Tom River, a tributary of the Ob [78]. The presence of HCH and DDT/DDE at low concentrations in the snow cover of Eastern Siberia was mainly associated with the regional atmospheric transport of OCPs still evaporating from agricultural soils since being banned in the USSR in the 1970–1980s [79].

Although MP concentrations at site Ob-D2 downstream of the Novosibirsk WWTP were significantly higher compared to those at the other two sites, the maximum particle-associated OCP and PCB content was observed for Ob-4 beach sands (Table 2). Interaction of MPs with POPs in surface waters and aquatic sediments depends on environmental conditions, polymer structure, shape, age, and even colour [22,80,81]. For example, the microparticles of PE, PP, PS, and PVC showed a high sorption potential for HCHs and PAHs and some other substances [82]; colourless MPs showed greater adsorption potential for PCBs compared to coloured ones [83].

Among the HCH isomers, only γ-HCH was detected on MPs from the Ob River beach sands at site Ob-D2, indicating the presence of fresh lindane contamination. In recent years, the circulation of HCH and DDT metabolites in biological systems has been demonstrated in various parts of the world [84,85,86,87,88]. The sorption capacity of PE, PP, and PS particles < 250 µm in diameter to α-, β-, γ-, and δ-HCH was proved experimentally [82]. Its relatively low concentration on MPs from the Ob beach sediments probably indicates non-periodic use or trace infiltration of this banned pesticide from potential pesticide burial sites. From the group of DDT metabolites, 4,4-DDT and 4,4-DDE were detected. The concentration of the latter prevails over the parent compound DDT, indicating the long-standing contamination of the environment with these pollutants. At the same time, the presence of the parent compound indicates a trace penetration of fresh toxicant into the Ob River ecosystem. It is also worth noting that pesticide concentrations are at trace levels and can be considered as “background”.

The situation is somewhat different for polychlorinated biphenyls. Of the low-chlorinated “light” PCBs, only one congener, 28, was detected, indicating the presence of fresh contamination at the time of sampling or a high fleet load in the Ob River. Highly chlorinated “heavy” PCBs were also detected, among which 118 and 180 dioxin-like congeners were present. Their concentrations per gram of MPs (up to 1274 and 3484 ng g^−1^, respectively) are high and may be harmful to the animals that ingest them. Moreover, 153 and 180 PCBs indicate long-standing contamination, the source of which may also be ships and small fleets on the river, or the high ability of plastic particles to adsorb PCBs, and 118 and 153 PCB congeners were previously detected in associations with MPs in marine debris [89] and beach sands [90]. In the latest study, the lowest levels of PCBs were found in rural beaches in Portugal, while the highest levels were found in urban beaches near large cities, highlighting the anthropogenic contribution to plastic pollution.

The detected concentrations of POPs were not frequent in the samples, but the detected concentrations of PCBs, DDT, and HCH significantly (from 1.2 to 12 times) exceed the levels found in MPs from urban beaches in Portugal [90,91], Bahía Blanca Estuary in Argentina [92], the coast of Japan, and the Pacific Ocean [93]. It should be noted that, in urban areas [90,91], the composition of PCBs was similar to what we found, which indicates an anthropogenic and technogenic impact [94]. At the same time, the levels of pesticides in the studied samples were also significantly higher, which may indicate both their higher concentration in the environment and the greater sorption capacity of the studied particles.

Polychlorinated biphenyls are among the most widespread POPs, due to their ubiquitous use in various areas of human life—from electrical equipment to carbon paper and paints [95,96]. Despite the complete ban on the production and use of PCBs worldwide [97], there are a number of “modern” sources of these compounds, closely associated with various thermal production processes, as well as e-waste pollution [94]. For example, a number of studies indicate that the cement and metal industries can make a significant contribution to PCB pollution [98,99,100]. Upstream of the study area (along the river course), there is an industrial zone with a number of cement factories and metal processing plants which apparently release PCBs into the local ecosystems. Thus, the increased concentrations of PCBs may indicate local pollution, while OCPs indicate global atmospheric transport and the long-term circulation of pollutants in the environment.

The investigation carried out is a pilot study and has some limitations, such as a low number of sampling points, the lack of proper monitoring, and the focus on recreational sites without testing the remote banks of the Ob River. It was also limited in its choice of POPs for evaluation, but future studies will extend the list to others such as PAHs. Based on the current study results, we cannot characterise the level of MP and POP pollution of the whole river. However, the study provides a springboard for further comprehensive research into the levels of MPs and POPs in river sediments and practical measures to tackle the problem.

## 5. Conclusions

The results of the current study obtained for the Ob River as an example indicate that Siberian water courses are exposed to MP and POP pollution. This study revealed associations of MPs with PCBs and OCPs that had not been previously detected in the Ob riverbed and beach sediments, indicating the circulation of these persistent substances in the river basin. MP concentrations in sand samples were significantly higher in number (*p* < 0.05) and mass (*p* < 0.01) downstream of the Novosibirsk WWTP outfall, suggesting its possible contribution to the pollution of the river compartments. OCPs, such as γ-HCH, 4,4-DDT and 4,4-DDE, and PCBs, including 118 dioxin-like congeners, were detected in pooled MP samples, reaching a total of 8937 ng g^−1^. The urban agglomeration of Novosibirsk and its satellite industries and agriculture may be the main contributors to pollution in the upper reaches of the Ob River. Despite the fact that the levels of most pollutants were quite low (except for PCBs 118 and 180), their ability to accumulate and the lack of elimination mechanisms provoke their accumulation in living organisms throughout life, which poses a danger both for individual organisms and for populations as a whole. The regular monitoring of the abundance and concentration of MPs and POPs in the Ob basin is needed to confirm and reliably identify the sources and effects of pollution.

## Figures and Tables

**Table 1:** MP counts and mass in the beach sands of the Ob River.

Sampling Site	Total MPs, Items	MPs m^−2^	MPs m^−3^	MPs kg^−1^
Ob-U3	8	32 ± 13.1	640 ± 261	0.41 ± 0.17
Ob-D2	26	104 ± 46.2	2080 ± 924	1.31 ± 0.58
Ob-4	6	24 ± 20.7	480 ± 413	0.3 ± 0.26
Mean	3.33 ± 2.9	53.3 ± 46.4	1067 ± 929	0.67 ± 0.58
	**Total MP Mass, µg**	**MP mg m^−2^**	**MP mg m^−3^**	**MP µg kg^−1^**
Ob-U3	120	0.48 ± 0.1	9.61 ± 2.06	6.11 ± 1.31
Ob-D2	304	1.22 ± 0.39	24.3 ± 7.73	15.3 ± 4.86
Ob-4	65.2	0.26 ± 0.21	5.22 ± 4.12	3.26 ± 2.57
Mean	40.8 ± 30.5	0.65 ± 0.49	13.1 ± 9.75	8.22 ± 6.13

**Table 2:** Content of different OCPs and PCBs in MP samples.

Sample	α- HCH	β- HCH	γ- HCH	δ- HCH	2,4- DDT	4,4- DDT	2,4- DDD	4,4- DDD	2,4- DDE	4,4- DDE	PCB 28	PCB 52	PCB 155	PCB 101	PCB 118	PCB 143	PCB 153	PCB 138	PCB 180	POP Sum
	ng g^−1^ of MPs																			
Ob-U3	* <0.3	<0.1	<0.3	<0.2	<0.6	<0.3	<0.2	<0.1	<0.3	<0.4	<0.6	<0.7	<0.1	<0.6	197.8	<0.5	<0.1	<0.2	1192	1390
Ob-D2	<0.3	<0.1	25.6	<0.2	<0.6	2504	<0.2	<0.1	<0.3	<0.4	<0.6	<0.7	<0.1	<0.6	215.4	<0.5	651.7	<0.2	501.7	3899
Ob-4	<0.3	<0.1	<0.3	<0.2	<0.6	<0.3	<0.2	<0.1	<0.3	3440	738.9	<0.7	<0.1	<0.6	1274	<0.5	<0.1	<0.2	3484	8937
	ng item^−1^																			
Ob-U3	<0.3	<0.1	<0.3	<0.2	<0.6	<0.3	<0.2	<0.1	<0.3	<0.4	<0.6	<0.7	<0.1	<0.6	1.0	<0.5	<0.1	<0.2	6.2	7.2
Ob-D2	<0.3	<0.1	0.04	<0.2	<0.6	4.3	<0.2	<0.1	<0.3	<0.4	<0.6	<0.7	<0.1	<0.6	0.4	<0.5	1.1	<0.2	0.9	6.74
Ob-4	<0.3	<0.1	<0.3	<0.2	<0.6	<0.3	<0.2	<0.1	<0.3	7.6	1.6	<0.7	<0.1	<0.6	2.8	<0.5	<0.1	<0.2	7.7	19.7

* <X—means limits of detection (LODs).

## Data Availability

The datasets analysed during the current study are available from the corresponding author upon reasonable request.
